# The Pathogenicity of BPI-ANCA in a Patient With Systemic Vasculitis

**DOI:** 10.3389/fimmu.2019.01334

**Published:** 2019-06-12

**Authors:** Sayo Takeda, Kanako Watanabe-Kusunoki, Daigo Nakazawa, Yoshihiro Kusunoki, Saori Nishio, Tatsuya Atsumi

**Affiliations:** Department of Rheumatology, Endocrinology and Nephrology, Faculty of Medicine and Graduate School of Medicine, Hokkaido University, Sapporo, Japan

**Keywords:** ANCA-associated vasculitis, neutrophil extracellular traps (NETs), Bactericidal/permeability-increasing protein (BPI), BPI-ANCA, immunity

## Abstract

**Objective:** ANCA associated vasculitis (AAV) is characterized by systemic necrotizing vasculitis with the presence of ANCA. Although BPI-ANCA is one of the atypical ANCAs and is occasionally seen in patients with vasculitis, the pathogenicity of BPI-ANCA remains unclear. This study was performed to examine the pathogenic role of BPI-ANCA against neutrophils.

**Methods:** A 76-year-old Japanese man showed BPI-ANCA positive systemic vasculitis with a medical history of *Pseudomonas aeruginosa* infection. BPI-ANCA IgGs were eluted from the patient serum using an immunoadsorbent column. *In vitro* experiment, healthy donor neutrophils were treated with BPI-AAV IgGs, MPO-AAV IgGs, healthy control IgGs under TNFα stimulation. After 3 h incubation, neutrophil extracellular trap (NET) was assessed by immunofluorescent imaging. To determine the pathogenicity of BPI-ANCA, TNFα-primed neutrophils were incubated with monoclonal BPI-ANCA in the presence or absence of recombinant BPI.

**Results:** BPI-AAV IgGs-treated neutrophils showed NET formation with histone citrullination. Interestingly, the monoclonal BPI-ANCA did not induce NET, but the immune complexes (ICs) of recombinant BPI and BPI-ANCA induced TNFα-dependent NET formation with hypercitrullination. Furthermore, TNFα increased the expression of BPIs in neutrophils and the BPIs were translocated to cell surface.

**Conclusion:** BPI-ANCA could affect neutrophils leading to NET formation and may play a role in the development of systemic vasculitis as pathogenic autoantibody.

## Key Message

1. BPI-ANCA activates neutrophil via forming immune complexes with BPI, leading to NET formation.
2. BPI-ANCA-induced NET may play role in the pathogenesis of vasculitis.
3. Gram-negative infections might be involved in the development of BPI-ANCA.

## Introduction

Bactericidal/permeability-increasing protein (BPI) is an antibacterial neutrophil cytoplasmic protein that plays an important role in the immune system. BPI has anti-endotoxin properties, which allow it to work against Gram-negative bacteria (GNB) infection. In addition, BPI delivers components of GNB to dendritic cells for processing. Anti-neutrophil cytoplasmic autoantibodies (ANCAs) against neutrophil granule BPI (BPI-ANCA) have been reported in various kinds of diseases, such as cystic fibrosis and inflammatory bowel diseases (IBD), in which opportunistic infections with GNB are common. During persistent GNB infections, complexes of BPI and GNB are processed and presented by dendritic cells, potentially leading to the breakdown of immunological tolerance against BPI (1). Although BPI-ANCAs are known to be involved in the pathogenesis of systemic vasculitis (2), it remains unclear whether they play a pathogenic role in the development of vasculitis. Here, we report that BPI-ANCAs in a patient with systemic vasculitis affect neutrophils to undergo neutrophil extracellular trap (NET) with hypercitrullination, thus contributing to the development of systemic vasculitis.

## Case Presentation

**Figure 1:**
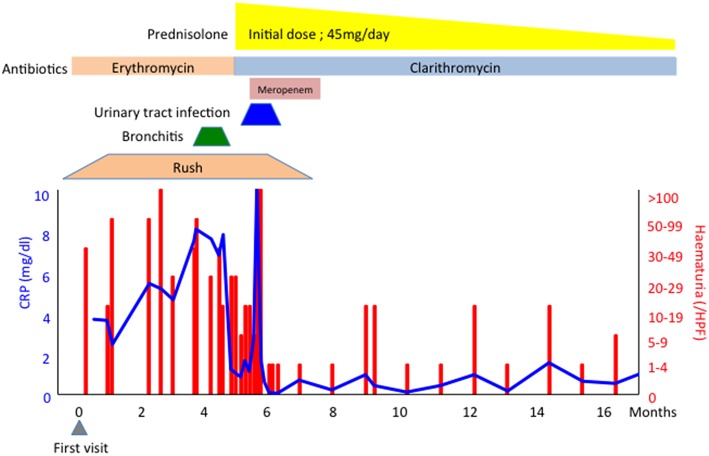
Clinical course of a patient.

A 76-year-old Japanese man who had experienced recurrent chronic bronchiolitis with *Pseudomonas aeruginosa* infection for over 10 years presented with a 6-month history of haematuria/proteinuria and purpura. A skin biopsy revealed cutaneous leukocytoclastic vasculitis, and a renal biopsy showed pauci-immune crescentic glomerulonephritis. Serum examination by immunofluorescence showed the patient was negative for P-ANCA, but positive for C-ANCA. Routine enzyme-linked immunosorbent assay (ELISA) revealed that he was negative for both MPO-ANCA and PR3-ANCA. The titer of serum immune complexes (C1q binding assay) was 50.0 μg/mL (normal range; < 3.0 μg/mL). Further ELISA assay (ANCA panel kit, Euro Diagnostica) revealed that the antigen for C-ANCA was BPI (titer; 6.5 O.D. ratio, Table 1) and other atypical ANCAs including azurocidin, cathepsin G, elastase, lactoferrin, and lysozyme were negative. Based on these findings, the patient was diagnosed with BPI-ANCA-associated systemic vasculitis (AAV). He was treated with prednisolone with antibiotics for GNB infections and his clinical findings were recovered (Figure 1).

**Table 1:** Laboratory data.

**Complete Blood Count**	
WBC	12,000 /μL
RBC	4.33 x 10^6^ /μL
Hemoglobin	11.2 g/dL
Platelet	29.4 ×10^4^ /μL
**Biochemistry**	
BUN	17 mg/dL
Cr	0.7 mg/dL
Cysc	1.43 mg/L
**Urinalysis**	
Haematuria	≥100 /HPF
WBC	5–9 /HPF
Proteinuria	0.76 UP/UCr ratio
**Immunoserological test**	
CRP	5.14 mg/dL
lgA	477 mg/dL
lgG	1,838 mg/dL
lgM	61 mg/dL
C3	42 mg/dL
C4	38 mg/dL
CH5O	11.0 U/mL
Immune complexes	50 μg/mL
(normal range; < 3.0 μg/mL)	
ANA	×160
RF	41.1 IU/mL
Anti DNA Ab	(–)
PR3 ANCA	<20.0 RU/mL
MPO-ANCA	<20.0 RU/mL
Anti GBM Ab	(–)
P-ANCA (IIF)	(–)
C-ANCA (IIF)	(+)
BPI ANCA	(+)/ 6.5 O.D.Value

*WBC, white blood cell; RBC, red blood cell; UP/UCr, urine protein to urine creatinine ratio; ANA, antinuclear antibody; RF, rheumatoid factor*.

### The Pathogenicity of BPI-ANCA in *in vitro* Experiments

**Figure 2:**
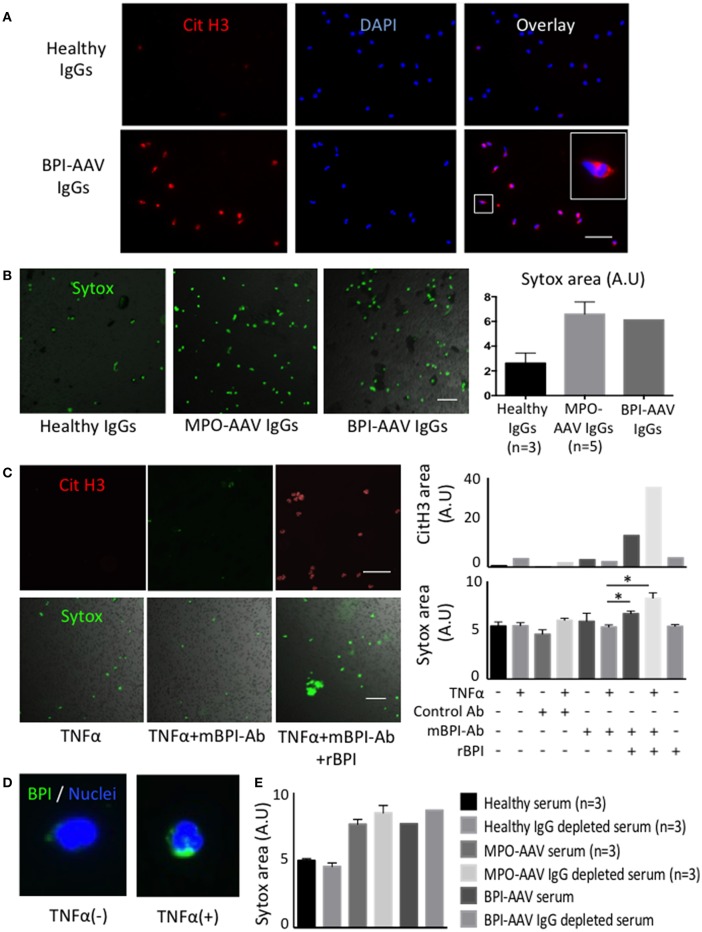
BPI-ANCAs induce NETs with hyper citrullinated histones. **(A)** Representative NETs staining of healthy or BPI-AAV patient IgGs-stimulated neutrophils using CitH3 (red) and DAPI (BLUE). **(B)** Representative Sytox-positive neutrophils live image. From the left, healthy IgGs (*n* = 3), MPO-AAV patient IgGs (*n* = 5), BPI-AAV patient IgGs. Right figure shows Sytox-positive areas quantified by Image J. **(C)** Representative CitH3-positive NETs and Sytox-positive neutrophils treated with TNFα, TNFα+mBPI-Ab, TNFα+mBPI-Ab+rBPI. Right figures show CitH3-positive area (upper) and Sytox-positive area (lower) under each situation. Data represent the mean±SEM of three independent experiments and were analyzed using unpaired *t*-test (PRISM software, GraphPad). ^*^p < 0.05. **(D)** BPI expression with or without TNFα stimulation. Green; BPI, BLUE; DAPI. **(E)** Sytox positivity of neutrophils treated with 3% serum, or 3% IgG depleted serum of patients with BPI-AAV, MPO-AAV (*n* = 3) and healthy control (*n* = 3). Scale bar in all figures, 50 mm. CitH3, citrullinated histone3; DAPI, 49,6-diamidin-2-phenylindol; BPI, bactericidal/permeability increasing protein; ANCA, anti neutrophil cytoplasmic antibody; AAV, ANCA associated vasculitis; IgG, immunoglobulin G; TNFα, tumor necrosis factor alpha; mBPI-Ab, monoclonal BPI antibody; rBPI, recombinant BPI.

To evaluate the significance of BPI-ANCAs in vasculitis, healthy donor neutrophils were treated *in vitro* with BPI-ANCA immunoglobulin (Ig)Gs derived from the patient. BPI-AAV IgGs-treated neutrophils underwent Sytox Green-positive NET formation with histone citrullination under TNFα stimulation in a manner similar to that of MPO-AAV IgGs-treated neutrophils (Figures 2A,B). Since the polyclonal ANCA-IgGs were extracted from patient sera using Protein G column, to elucidate the pathogenicity of BPI-ANCA, a TNFα-primed neutrophils were treated with monoclonal BPI-ANCA and control antibody in the presence of recombinant BPI. Although the monoclonal BPI-ANCA did not induce NET formation, the immune complexes (ICs) of recombinant BPI and BPI-ANCA induced TNFα-dependent NET formation with hypercitrullination (Figure 2C). To elucidate the phenomenon that TNFα accelerated the ICs-induced NET formation, the expression of BPI in neutrophils with or without TNFα stimulation was evaluated by immunostaining. TNFα up-regulated BPI expression in neutrophils and the overexpressed BPIs were translocated to cell surface (Figure 2D).

## Discussion

We demonstrated that BPI-ANCAs in a patient with systemic vasculitis activated neutrophils to undergo NET formation *in vitro* studies, suggesting that BPI-ANCAs contribute to the pathogenicity during the development of vasculitis.

BPI-ANCAs have been frequently detected in various kinds of disease, including cystic fibrosis and inflammatory bowel diseases, and these auto-antibodies were found to be involved in organ damage and excessive inflammation (1). Meanwhile, Zhao et al. reported that BPI-ANCAs were detected in 40% of patients with double-negative ANCA (negative for MPO- and PR3-ANCA) vasculitis (2). Although MPO- and PR3-ANCAs are known as pathogenic antibodies that can activate neutrophils (3, 4), the role of BPI-ANCAs remains unclear.

Here, we found that BPI-ANCA IgGs activated neutrophils under TNFα stimulation, leading to NET formation with hypercitrullination. Furthermore, monoclonal BPI-ANCA induced NETs with hyper-citrullination via forming ICs of BPI and BPI-ANCAs in accordance with TNFα stimulation. The C1q binding ICs were detected by ELISA method in this patient (Table 1), implying that the patient-eluded BPI-ANCAs may form ICs with BPI in the absence of additional exogenous BPIs and IgGs themselves could have NET inducibility. In addition, BPIs on neutrophils were translocated to cell surface and overexpressed by TNFα stimulation. These phenomena indicate that BPI-ANCAs may be produced during chronic inflammatory diseases, such as cystic fibrosis and IBD, and consequently (1) BPI-ANCAs bind to circulating excessive BPIs that are exposed by infections, inducing the formation of ICs, (2) the circulating ICs may bind to neutrophils with overexpressed BPI, (3) TNFα facilitated ICs mediated signaling such as spleen tyrosine kinase (5), leading to NET formation, and (4) these contribute to the development of systemic vasculitis.

In addition, Traaij et al. (5) reported that ANCA-IgG-depleted serum induced NET formation and that the NET inducibility was associated with the vasculitis activity. Therefore, we conducted NET experiments using both whole serum and IgG-depleted serum. In contrast with healthy samples, the serum and IgG-depleted serum from patients with either MPO-AAV or BPI-AAV induced NET formation (Figure 2E), suggesting that some humoral factors as well as BPI-ANCAs might influence NET formation and the pathogenesis of disease.

Although most patients with cystic fibrosis have BPI-ANCAs, it remains controversial why only a few patients of BPI-ANCAs positive develop systemic vasculitis. There are several possible explanations, including the amount of exposed BPI during infection, the presence of refractory infection, and the characteristics of BPI-ANCAs (e.g., affinity to antigen and IgG subclasses). While, BPI-ANCAs IgG derived from patients in this study.

## Concluding Remarks

This study showed that BPI-ANCAs could influence NET formation and may play a role in the development of systemic vasculitis as pathogenic autoantibodies.

## Materials and Methods

### Patients and Healthy Controls

Blood samples were obtained from the patient with BPI-AAV, MPO-AAV and healthy control. This study was approved by the Institutional Review Board of Hokkaido University Hospital. We obtained written informed consent for the publication from the patient.

### Nets Induction Assay

Neutrophils were extracted from a peripheral blood sample of a healthy volunteer using Polymorph Prep. After 30 min of 5 ng/mL tumor necrosis factor (TNF)α pretreatment at 37°C, the neutrophils were treated with 3% serum, or 3% IgG depleted serum, or 400 μg/mL IgGs eluted from serum of patients with BPI- AAV, MPO-AAV, healthy control. In experiment using monoclonal antibody, mouse BPI monoclonal antibody (10 μg/mL) and recombinant BPI (Santa Cruz) were used. The neutrophils were seeded on chamber slides or 96-well plate and incubated for 3 h at 37°C *in vitro*.

IgGs were eluted from serum using an immunoadsorbent column (Protein G HP SpinTrap; GE Healthcare, Tokyo, Japan) according to the manufacturer protocol. Contamination of endotoxin in samples was ruled out using the Limulus test kit (Wako Pure Chemical, Osaka, Japan). NETs were evaluated by immunofluorescent staining and by live imaging.

### Immunofluorescent Staining for NETs and Neutrophil BPI

After incubation, neutrophils were fixed by 4% paraformaldehyde (PFA). After blocking (2%BSA for 30 min at room temperature), citrullinated histone 3 (CitH3) antibodies (rabbit IgG, 1:200, dilution, for 24 h at 4°C, Abcam) and BPI antibody (mouse IgG, 1:100, for 24 h at 4°C, Santa Cruz) were used. DNA was stained using DAPI.

### Live Imaging *in vitro*

To evaluate neutrophil death including NETs, sytox green reagents (final concentration = 15 nM, Thermo Fisher Scientific) were applied to neutrophils and Sytox-positive neutrophils and NETs were visualized and quantified by Fluorescence microscopy (Keyence). The CitH3-positive NETs and Sytox-positive NETs/dead neutrophils were quantified using Image J software.

## Ethics Statement

All procedures performed in the study were in accordance with the Declaration of Helsinki. Written informed consent was obtained from the patient presented here.

## Author Contributions

ST and DN wrote and summarized the manuscript. KW-K and DN conducted *in vitro* neutrophil experiments. ST, KW-K, YK, SN, and TA managed and followed up the patients' care. All authors reviewed the manuscript and approved the final version.

### Conflict of Interest Statement

The authors declare that the research was conducted in the absence of any commercial or financial relationships that could be construed as a potential conflict of interest.
